# The Role of English as a Foreign Language Teachers’ Mindfulness and Compassion in Fostering Students’ Foreign Language Enjoyment

**DOI:** 10.3389/fpsyg.2022.899298

**Published:** 2022-05-03

**Authors:** Jingjing Huang

**Affiliations:** School of Foreign Languages, Hefei Normal University, Hefei, China

**Keywords:** positive psychology, teachers’ mindfulness, teachers’ compassion, students’ enjoyment, EFL

## Abstract

With the popularity of positive psychology in English as a Foreign Language (EFL) teaching and learning, learners’ positive emotions have attracted great academic attention. Foreign language enjoyment (FLE) is regarded as a constructive emotion and key component for learners’ academic engagement that is affected by educators’ emotions and psychological attributes. Earlier studies have proved the positive role of educators’ mindfulness and compassion in reducing learners’ negative feelings, boosting their positive emotions and building a harmonious teacher-student rapport. Through mindful and compassionate training, EFL teachers are skilled at creating a joyful learning atmosphere, showing understanding and support toward learners, as well as inspiring learners with enthusiasm and joy. The present review makes efforts to emphasize the significant effect of EFL teachers’ mindfulness and compassion on fostering students’ FLE. Moreover, a number of practical implications are provided for EFL teachers, teacher educators, school managers, and future directions are offered for enthusiastic researchers to conduct similar and complementary research in the field of foreign language education.

## Introduction

With the shift of research focus from negative psychology to positive psychology both within and outside the language education field (Seligman, 2011; Dewaele and MacIntyre, 2014; Mercer and Dörnyei, 2020), researchers have adopted a holistic perspective toward second language learning, arguing English language teaching should focus not only on the transfer of language knowledge and the training of language skills, but also on the expression of both teachers’ and students’ emotions (MacIntyre and Gregersen, 2012; Boudreau et al., 2018; Yin et al., 2019; Dewaele and Li, 2021). From the perspective of positive psychology, language educators aim to seek ways to reduce negative feelings like burnout, anxiety and boredom (Schaufeli et al., 2009; Dewaele, 2013; Pawlak et al., 2020), as well as promote positive emotions and traits, such as enjoyment, optimism, mindfulness, compassion, efficacy and well-being (Dewaele et al., 2017; Oxford, 2016; Fathi and Derakhshan, 2019). Positive emotions broaden people’s thought-action repertoires, build enduring resources for the future, undo the detrimental effects of negative emotions (Fredrickson, 2013), and engender behaviors such as play, creativity, curiosity, and exploration, which are widely seen as beneficial in learning (Boudreau et al., 2018). Many researchers claim EFL teachers play a critical role in creating a supportive and enjoyable learning environment that is of vital significance for learning results, and a considerable number of studies have approved the influences of EFL teacher relational elements upon students’ emotions and academic performances (Keller et al., 2018; Delos Reyes and Torio, 2020; Fathi et al., 2021; Pishghadam et al., 2021). Effective educators provide learners with language input, engage students in the lesson, coordinate interpersonal relationship, give feedback to leaners’ language products, and promote learners’ long-term language achievement (Coombe, 2014). Language educators are aware of the importance of improving students’ experience of language learning by enabling them to develop and maintain positive emotions (MacIntyre and Mercer, 2014), and they fuel students’ enjoyment and engagement by fostering a friendly atmosphere with low anxiety (Dewaele et al., 2017). Within a favorable language learning environment, students can better process everything in the academic situation, absorb more language input and generate more positive feelings, leading to success and achievement (Fang and Tang, 2021).

As one of the frequently knowledgeable emotions in language learning field, foreign language enjoyment (FLE) is a positive, stimulating and activity focused emotion (Piniel and Albert, 2018). Numerous studies have shown that FLE enhances learners’ pleasure in second language learning, increases their willingness to communicate and helps them acquire self-perceived and actual language proficiency (Boudreau et al., 2018; Khajavy et al., 2018; Li et al., 2020). Being a positive emotional state that relates to challenge, happiness, passion, pride and satisfaction, classroom enjoyment is perceived as a way to expand students’ perspectives and enhance their cognitive abilities through the process of language learning (Dewaele and MacIntyre, 2014). Recent research has demonstrated the supportive role EFL teachers play in fostering students’ FLE. Although learning a foreign language is usually related with negative emotions, good educators are skilled at reducing learners’ psychological stress and cultivating an enjoyable and positive learning environment (Ahmadi-Azad et al., 2020). Similarly, Dewaele et al. (2017) discovered that effective instructors pay greater attention to their students’ passion and enjoyment by providing a friendly and harmonious classroom atmosphere with low anxiety levels. As Piniel and Albert (2018) reported, it is possible for educators to be aware of a wide array of emotional states students experience, and improve teaching effect through promoting enjoyment in the classroom, especially when they choose the topics students like and are positive about. The dyadic teacher-student interaction, mediated by teachers’ intentional facial expressions and gestures showing care and understanding, is considered the primary mechanism of bolstering FLE in the classroom (Talebzadeh et al., 2019). Dewaele et al. (2019) also investigated the association between a variety of teacher-centered variables and FLE in a Spanish classroom context. Teachers should be mindful to recognize the significance of invoking students’ enjoyment, by way of building caring relationships with students as well as providing various interesting and challenging activities to engage students (Pavelescu and Petric, 2018).

Currently, a great deal of discussion has been going on about what qualities make a good teacher, and there is growing interest in exploring teachers’ mindfulness in effective teaching (Roeser et al., 2012). The construct of mindfulness is defined as an awareness that arises as a result of intentionally attending in an open, caring, and discerning way (Shapiro and Carlson, 2009). The integration of mindfulness into teaching has tremendous benefits for both students’ positive emotions and academic achievement, helping students enhance self-regulatory capacities and interpersonal skills, promote creativity, resilience, well-being, and other psychological strengths which may contribute to healthy learning climates (Shapiro et al., 2011; Ergas and Hadar, 2019). Mindful teachers are calm, clear and kind in the classroom context, indicating an integration of stable psychological state, clarity of words and deeds, as well as harmonious interaction with students, which provides students with emotional support and learning autonomy (Rickert, 2016). In allowing themselves as well as their students to experience feelings of acceptance and harmony, teachers demonstrate to students that they are understood and are in a safe environment, resulting in physiological relaxation and mental openness, thereby creating joyful mutual responsiveness (Geller and Greenberg, 2012). Thus, mindfulness training can be particularly beneficial for teachers as a means of managing emotions and facilitating the development of awareness skills. Through mindfulness practices, educators become conscious of their own physical and psychological experiences, and are better able to give personal responses to learners’ behaviors in a positive light, leading to improvements in their relationships with students (Shapiro et al., 2016). In addition, educators with higher level of mindfulness are more likely to practice perspective-taking and apply proactive class management strategies than reactive techniques to tackle with disciplines, as well as show greater empathy and be better positioned to help learners cultivate positive emotions, naturally causing learners to become engaged and feel enjoyment in learning (Jennings, 2014).

Moreover, through mindfulness practice, the development of compassion begins with learning to relate to oneself with greater acceptance and kindness (Shapiro and Carlson, 2009). They defined compassion as a combination of two qualities: the ability to feel empathy for the suffering of oneself or others, along with the desire to act to alleviate the suffering. As Eldor and Shoshani (2016) stated, in the teaching context, compassion is an expression of educators’ attitude toward both learners and other adults such as their teaching colleagues, and can therefore be a potential source of emotional vigor, organizational commitment and job satisfaction, helping them prevent negative feelings like burnout as well as generate positive feelings of well-being and efficacy. Self-compassion is considered a constructive factor to help English language teachers reduce job stress and professional burnout, fostering job-related affective well- being (Rajabi and Ghezelsefloo, 2020). Additionally, compassion manifests itself in classroom by a caring and respectful relationship between teachers and students (White, 2017). On one hand, students desire to be cared for, respected and valued, arguing that teachers’ understanding and acceptance can help them enjoy the learning process and feel satisfied with school (Anderson and Graham, 2016). On the other hand, compassionate and friendly teachers are viewed as more likely to respond to students’ voices and take more time to support students, motivating students to do well (Koutselini, 2017). Educators who are positive and respectful of learners can facilitate classroom enjoyment by showing compassion toward their mistakes even with a sympathetic laughter (Dewaele et al., 2017). It is recommended that increasing the compassion levels of the teachers will have a positive effect on fostering teacher-student rapport and reaching the desired goals of education (Çalışoğlu, 2018).

Foreign language enjoyment could be simultaneously enhanced by learners’ private pleasure, by educators’ recognition and attitude, and by the positive classroom atmosphere (Li et al., 2018). The previous inquiries were conducted with respect to the association of FLE with a lot of teachers’ emotions and behaviors (Dewaele et al., 2017; Piniel and Albert, 2018; Talebzadeh et al., 2019). In addition, a bulk of study has proved the constructive role of mindfulness and compassion in reducing negative feelings, boosting positive emotions and creating caring and harmonious interpersonal relationships in various situations (Shapiro and Carlson, 2009; Frank et al., 2015; Conversano et al., 2020). It makes sense that mindful and compassionate educators take time and support to understand learners’ perspective, and continue making pedagogical choices that help students enjoy second language learning. However, to the best of the researcher’s knowledge, research about the effect of teacher’s mindfulness and compassion on students’ foreign language learning is scant, and no studies to date have explored their association with the positive emotion of enjoyment. Therefore, this review aims to consider the function of teachers’ mindfulness and compassion on FLE.

## Review of Literature

### Mindfulness

The term mindfulness originates from the Pali word sati combined with sampajañña, signifying “the presence of mind and attentiveness to the present” (Bodhi, 2000). Despite the complexity of defining mindfulness, many previous studies have utilized Jon Kabat-Zinn’s (1994) definition of mindfulness as paying attention in a particular way: on purpose, in the present moment, and nonjudgmentally. Guided by the teaching that mindfulness is both a process and an outcome, Shapiro and Carlson (2009) described mindfulness as both mindful awareness and mindful practice. The former is a natural human capacity, a knowing and experiencing of life, allowing us to see clearly which experiences lead to greater benefits or suffering for ourselves and others; while the latter refers to a systematic training that consists of three core elements: intention, attention, and attitude (Shapiro et al., 2006), involving the conscious development of skills such as ability to sustain attention, acceptance, discernment and compassion. Mindfulness allows for the creation of autobiographical meaning in a more flexible manner, thereby enhancing human capacity to constructively reassess adverse experiences and relish positive ones (Garland et al., 2015). Multiple studies have demonstrated that mindfulness practice promotes wellbeing, improves interpersonal relationships, alleviates stress and prevents burnout (Bernay, 2014; Hue and Lau, 2015; Kerr et al., 2017).

Within the field of education, Ergas and Hadar (2019) identified two dimensions of mindfulness: mindfulness in education, revolving around interventions with concrete measurable effects in physical-mental health, cognitive function and emotional regulation; and mindfulness as education, serving as a means for scaffolding education, characterized by contemplative pedagogy, life-long learning and sporadic whole-school implementations. It is generally accepted that mindfulness can be trained and enhanced to generate positive emotions. Over the past decades, the most widely used mindfulness-based intervention is mindfulness-based stress reduction (MBSR) (Kabat-Zinn, 2009), which is proved to be effective in regulating emotion and enhancing attentional skills by rigorous studies. Gold et al. (2010) reported that an 8-week MBSR course has a positive impact on primary teachers’ self-confidence, self-efficacy, problem solving and taking time to relax, while reducing anxiety, pressure and depression. According to Flook et al. (2013), mindfulness and its impact on educators can be inter-related. They reported a modified MBSR training tailored for teachers shows significant effect on reduction in their psychological symptoms, improvements in classroom management and increases in self-compassion. Teaching involves the ability to communicate, to establish relationships with students, to motivate them, and to create a pro-social classroom (Jennings and Greenberg, 2009). Therefore, by incorporating contemplative approaches to mindfulness in the classroom, teachers’ social and emotional competence is enhanced, resulting in the creation of a more harmonious, united classroom with improved relationships with students, classroom management and student performance (Jennings and Greenberg, 2009; Roeser, 2016). Moreover, mindfulness skills are applied by teachers during curriculum development and implementation to facilitate positive changes in the classroom, like helping students center attention and engaging student in active learning (Napoli, 2004). As Jennings (2015) declared, mindfulness helps teachers be aware of their own emotions and tendencies, knowing how to use emotions such as joy and enthusiasm to motivate students’ learning; mindfulness also cultivates teachers’ social awareness to understand how their behavior and emotion affect their interaction with students, thus helping them build supportive and encouraging relationships with students as well as find effective solutions to conflict, so as to trigger students’ natural tendency to enjoy learning. The present literature shows that although mindfulness has been researched in terms of conceptual definition, its relationship with emotions and application in various groups like preservice teachers, early childhood teachers and primary school teachers, little exploration has been carried out to discover the effects of mindfulness in second language learning and teaching.

### Compassion

Deriving from the Latin cum patior, the word compassion means suffering, undergoing, and standing in solidarity with others (Straub, 2000). The conception of compassion has been investigated from various perspectives such as philosophy, psychology, ethics, and biology (Seppelä et al., 2017; Kim et al., 2020). As Gilbert (2014) stated, it is a behavioral process involving many compassionate activities relevant to understanding the source of pain and the desire to reduce other people’s suffering. Sprecher and Fehr (2005) described compassion as a kind of sensitive love, with an emotional, cognitive, and behavioral motivation to support and help others in times of depression. Despite its intricate nature, it is commonly agreed that compassion is an affective state that is action-oriented, including the following four characteristics: an awareness of other’s suffering and psychological state; a feeling of kindness; a desire to alleviate other’s suffering; and acting to reduce that suffering (Goetz et al., 2010). Similarly, Neff (2015) interpreted compassion as a boundless mental and emotional state that is accessible to everyone by virtue of being human, which generates our capacity for love, wisdom, courage, and generosity as well as helps us sustain the act of caring for others.

Compassion is also regarded as a skill can be taught and cultivated through learning and practice. Jazaieri et al. (2013) reported a 9-week mindful compassion cultivation training program helps participants increase the ability to engage in self-compassion and compassion for others, and thus significantly increasing their mindfulness and happiness, as well as reducing anxiety and emotional depression. In clinical psychology, a compassion focused therapy has been proved to be positively connected with affiliative experiences, which in turn foster our courage to face and engage with difficulties, thereby motivating us to function at our optimum (Gilbert, 2014). Similarly, a mindfulness-based compassion practice has been shown to importantly reduce participants’ stress and increase their attention, resilience and cognitive regulation (Bresciani Ludvik, 2016). According to Kernochan et al. (2007), mindfulness training also leads to compassion due to its focus on the moment, including others’ and their own suffering. In such a state of equanimity, our compassion is naturally elicited and our attention is redirected from our own desires to those of others.

In the educational sector, compassion, as an active attitude and action for change, could also be cultivated in classroom to associate teachers’ role with their students’ emotional and social well-being, so it is suggested teachers should shift from teaching instrumental knowledge to cultivating the whole person, from alienation to inspiration, resulting in the self-actualization of both teachers and students (Koutselini, 2017). Rajabi and Ghezelsefloo (2020) investigated the correlation between job stress and affective well-being as moderated by self-compassion with its subscales such as self-kindness, common humanity, and mindfulness, confirming the application of self-compassion among Iranian English language teachers can be effective in reducing stress and improving self-esteem, optimism, joy and happiness. Moreover, Çalışoğlu (2018) conducted a study to analyze teachers’ compassion levels in terms of different variables, such as gender, age, marital status and working place, and suggested it is of high importance to provide teachers with various in-service trainings to improve their compassionate levels. Given the essential link between compassion and education, it is imperative for educators to focus more on learners’ emotion, allow learners to share their views, show understanding toward learner’ shyness as well as encourage them to participate in the well-designed class activities. The implementation of educators’ compassionate actions provides learners with a safe and trusting platform, and serves to enhance learners’ experience, giving them motivation and joy, as well as fostering their progression in learning (Gill and Ursuleanu, 2017). While the notion of compassion has attracted the attention of many education researchers, any exploration on its relevance to EFL domain remains limited.

### Enjoyment

Enjoyment is a complex emotion that encompasses many dimensions of challenge and perceived ability, all of which reflect the human drive to succeed when faced with difficult trials (Csikszentmihalyi, 2008, 2014). It is characterized by a sense of accomplishment arising from completing a complex task that stimulates further investigation (Pekrun et al., 2007), and comes with successful performance, sustaining persistence and enthusiasm (Ainley and Hidi, 2014). Enjoyment occurs when people not only satisfy their needs, but also go beyond their previous expectations to achieve something unexpected or unimagined, and it is an essential component of flow experience that fosters holistic engagement and involvement in a challenging activity at an optimal level (Csikszentmihalyi, 2008).

According to the broaden-and-build theory (Fredrickson, 2001), the positive emotion like enjoyment contributes to expanding experience and acquisition of adaptive knowledge. Taking a broaden-and-build perspective on positive emotion, Dewaele and MacIntyre (2014) introduced the concept of enjoyment into the foreign language learning field and developed a 21-item Foreign Language Enjoyment Scale, pointing out FLE can be measured by various facets, such as creativity, pride, interest, fun, and positive class environment. Hence, it is logical to expect that students experience enjoyment when they are allowed a certain freedom in exchange, receive recognition from teachers, or do challenging and creative activities. They further confirmed a two-factor structure of the FLE Scale including FLE-Private and FLE-Social: the former refers to positive feelings about realizing one’s own progress and achieving good results in foreign language learning, while the latter is motivated by encouraging peers and teachers as well as a supportive environment (Dewaele and MacIntyre, 2016). Since learners may appreciate FLE in many aspects, Li et al. (2018) have validated three new factors of FLE: the FLE-private factor, positive feelings about fun and success in learning; the FLE-teacher factor, highlighting EFL teachers’ role in creating a joyful atmosphere and applying pedagogical skills, such as being compassionate and supportive toward learners, allowing them learning autonomy and fostering their engagement; and the FLE-atmosphere factor, referring to the classroom environment where the self, peers and teachers are all engaged and form a positive whole so as to reinforce enjoyment and foster closer social bonds. Moreover, grounded on the control-value theory, FLE is an activating emotion focusing on activity and has been demonstrated to exert a positive impact on learners’ academic achievement (Pekrun et al., 2007; Piniel and Albert, 2018). It is worth noting that FLE leads to action tendencies that are different from negative emotions, empowering learners to utilize their positive potential, improve their acquisition of EFL content, overcome the annoying effects of negative feelings and enhance their persistence through times of setbacks (MacIntyre and Gregersen, 2012; Saito et al., 2018).

Recent literature has shown that educators play a supportive role in the development of learners’ FLE. Dewaele et al. (2017) declared teachers pay great attention to facilitating their students’ FLE, and there exists a strong relationship between what teachers actually do in class and the extent to which students enjoy the foreign language learning, so teachers should focus on making their classes enjoyable by giving students choices in learning, using humor and praise appropriately, as well as apply sympathetic interventions when things go wrong. By creating an appropriate learning climate by providing continuous feedback, assigning tasks of varying competence levels and allowing cooperative learning, educators are able to support learners’ experiences of enjoyment and achievement (Hagenauer and Hascher, 2014). Besides, there is a transmission of enjoyment from teachers to students. Enjoyment contagion occurs when teachers display interest and enthusiasm in teaching, and try to establish a warm and caring relationship with students. Teacher’s happiness in the foreign language class is positively transmitted to students, linked to students’ motivation toward learning as well as their attitude toward teacher (Moskowitz and Dewaele, 2021). Since emotionally positive learning possibly leads to academic achievement and success, several inquiries were carried out with respect to FLE, but the variable factors contributing to FLE still wait to be further investigated.

## Implications and Future Directions

The research of positive psychology in second language acquisition is paramount to exploring and nurturing constructive emotions, traits and skills in language teaching field. The present review has provided some suggestions for not only language educators but also other language stakeholders like teacher educators, institution managers, policymakers, or researchers. It intends to influence educational psychology and contributes to the literature regarding the value of educators’ positive associations with learners in the teaching environment. A learner with greater language enjoyment is more likely to have more engagement that contributes to greater academic success. The degree to which learners enjoy the foreign language class is influenced by the actions and attitudes of the educators, the organization of the class, and more specifically, the teacher-student rapport, so good educators who can create positive atmosphere enhance learners’ willingness to improve their skills and are appreciated by learners (Dewaele and MacIntyre, 2014). Previous research indicates that the integration of mindfulness and compassion into education can be extremely beneficial, both in boosting students’ positive emotions and in building an enjoyable learning atmosphere. Thus, there is an urgent need for academic institutions to pay attention to the constructive attributes of educators and learners.

Since emotions are strongly related to various cognitive dimensions and educational performance according to studies conducted before, it is imperative for educators to be conscious of learners’ emotions and the learning climate. For EFL teachers, the current review suggests that they should take into consideration learners’ emotional reactions, and assist them in minimizing their negative feelings like anxiety, boredom and hopelessness, as well as maximizing the positive emotions like enjoyment, enthusiasm and well-being. Teachers can take different actions such as taking care of their talk, being careful about feedback to students, listening to students, employing questions to engage students, and rethinking classroom management as managing relationships (Mercer and Dörnyei, 2020). EFL teachers should first cultivate mindfulness and compassion as stable personality traits, consciously discerning students’ needs and responding in a proper and skillful way, as well as nurturing affective emotions, such as friendliness and understanding, in relationship to students. Being mindful involves being accepting, attentive, and warm; being compassionate includes being supportive and warm (Kernochan et al., 2007). The role of mindful, reflective and compassionate teachers is not just limited to the transmission of linguistic and content knowledge to students. But more importantly, EFL teachers take responsibility for providing a positive learning climate both in and out of class, managing the emotional atmosphere of the classroom, establishing a harmonious relationship with students, and ideally, inspiring students with passion and joy. Besides, EFL teachers should also be aware of their own emotions, get rid of the control of negative emotions, and endeavor to foster self-compassion and well-being, for emotions are transmittable, and teachers’ positive emotions are important indicators of FLE mentioned by students. When mindfulness and compassion are integrated into teaching, both EFL teachers and students will enjoy more, and understand the interactive connections and attachments they share. With the constructive and expressive teacher-student relationship, teachers offer their students important psychological assets such as enjoyment, security, hope and optimism (Carmona-Halty et al., 2019). It is the teacher’s concern to show compassion and understanding toward students so as to build a safe, motivating and encouraging class where students are truly engaged and experience enjoyment in learning.

This field of study is also useful for teacher educators who must be aware that mindfulness and compassion training aids teachers in reducing the negative effects of unpleasant feelings and enhancing positive emotions. Mindfulness and compassion may make valuable contributions to the social and emotional competencies teachers need to create and maintain an emotionally supportive classroom (Jennings, 2014). Thus, teacher training courses must take the emotional aspects of educators into account, aiming to amplifying their pleasant feelings and professional fulfillment. The mindfulness-based interventions help teachers not only reduce job-related stress and burnout, but also improve well-being (Roeser et al., 2012; Roeser, 2016), so teacher educators should design training programs with practical techniques to apply the practice of focused attention, compassion toward oneself and others, and coping strategies for work anxiety and stress for EFL teachers. The training should emphasize developing mindful awareness and self-regulation of thoughts, emotions, and behavior, in addition to using facilitated mindfulness and compassion practices to utilize these skills and attitudes in teaching and interacting with students. Subsequently, by maintaining an intentional awareness and understanding toward others’ suffering, EFL teachers are more likely to focus on students’ needs and emotions, making use of mindfulness and compassion as constructive contributors to provide a safe, nurturing and relaxing learning environment. Furthermore, the training programs and workshops allow teachers to acquire the skills of helping students improve their mindfulness and compassion level so that students can be conscious of their aptitudes and capabilities, focus attention in class, perceive happiness actively, face difficulties with positive attitudes, and learn to accept imperfectness, all of which may contribute to fostering enjoyment while learning a foreign language.

In addition, the outcome of the research may help school managers in particular regard educators’ emotional and psychological aspects as important means to ensure learners’ academic enjoyment and achievement. With mindfulness and compassion, educators can strengthen their self-attunement and are able to relate to themselves with greater acceptance and understanding, inspiring a natural tendency to reduce the adverse effects of unpleasant feelings and savor the positive aspects of experience (Shapiro and Carlson, 2009). Hence it is advisable for school managers to offer relevant courses to promote EFL teachers’ mindfulness and compassion at the beginning of their qualified occupations to guarantee their pleasant feelings and job satisfaction. Moreover, it is important for school managers and policymakers to upgrade educational conditions like providing teacher training programs, introducing advanced teaching resources and facilities, in order to build a healthy, optimistic and constructive school environment. These perspectives further propose that students feel enjoyment in foreign language learning when mindful and compassionate EFL teachers feel happy and optimistic in the workplace, become aware of their obligation in supporting and engaging students, as well as show caring and understanding toward students.

The present study provides some proof that educators’ mindfulness and compassion are possibly associated with learners’ enjoyment in learning. While the previous study puts emphasis on discovering the impact of teachers’ mindfulness and compassion on their behavior and emotional regulation, how they are correlated with students’ FLE and learning experiences should not be ignored. Thus, future study can be conducted in this domain to explore the relationship between students’ FLE and teachers’ mindfulness along with compassion in educational contexts. Besides, this review mainly focuses on the three psychological and emotional factors of mindfulness, compassion and enjoyment, more studies should emphasize other variables like interest, involvement, or loving pedagogy, as indicators of students’ FLE, or investigate the cause-and-effect relationship of teachers’ mindfulness and compassion with other variables like optimism, well-being, grit, and emotional intelligence, as well as explore the interplay of several variables and its effect in language education. Moreover, it is recommended that more empirical studies with a mixed methods approach combining quantitative data with qualitative analysis or longitudinal studies can be conducted.

## Ethics Statement

The studies involving human participants were reviewed and approved by Hefei Normal University Academic Ethics Committee. The patients/participants provided their written informed consent to participate in this study.

## Author Contributions

JH confirms being the sole contributor of this work and has approved it for publication.

## Conflict of Interest

The author declares that the research was conducted in the absence of any commercial or financial relationships that could be construed as a potential conflict of interest.

## Publisher’s Note

All claims expressed in this article are solely those of the authors and do not necessarily represent those of their affiliated organizations, or those of the publisher, the editors and the reviewers. Any product that may be evaluated in this article, or claim that may be made by its manufacturer, is not guaranteed or endorsed by the publisher.
